# *ADCY5* ‐Mosaic Variants: A Diagnosis Not to Be Missed

**DOI:** 10.1002/mdc3.70175

**Published:** 2025-06-12

**Authors:** Alice Innocenti, Emmanuel Roze, Florence Riant, Marie‐Céline François‐Heude, Bérénice Lecardonnel, Giacomo Garone, Maxime Colmard, Eline Chauvet‐Piat, Anne‐Cécile Chaux, Marie‐Aude Spitz, Beatrice Desnous, Catherine Sarret, Philippe Damier, Thomas Wirth, Mathieu Anheim, Emilie Retailleau, Estelle Conabady, Caroline Dubacq, Oriane Trouillard, Aurélie Méneret, Agathe Roubertie

**Affiliations:** ^1^ Neurology, Epilepsy and Movement Disorders Unit Bambino Gesù Children's Hospital, IRCCS, Full Member of European Reference Network on Rare and Complex Epilepsies, EpiCARE Rome Italy; ^2^ Département de Neurologie Hôpital Pitié‐Salpêtrière Paris France; ^3^ Institut du Cerveau et de la Moelle Épinière, ICM Sorbonne Université, INSERM, CNRS, APHP, Hôpital de la Pitié Salpêtrière Paris Paris France; ^4^ Service de Génétique Moléculaire Neurovasculaire Hôpital Saint Louis Paris France; ^5^ Service de Neuropediatrie CHU Montpellier Montpellier France; ^6^ Service de EEG Pédiatrique CHU de Saint‐Etienne Saint‐Etienne France; ^7^ Service de Neuropédiatrie Hôpitaux Universitaires de Strasbourg Strasbourg France; ^8^ Service de Neuropédiatrie APHM, Timone Enfant Marseille France; ^9^ Services de Pédiatrie et Génétique Médicale Hôpital Estaing, CHU de Clermont‐Ferrand Clermont‐Ferrand France; ^10^ Service de Neurologie Nantes Université, CHU de Nantes, CIC1413 Nantes France; ^11^ Service de Neurologie Hôpitaux Universitaires de Strasbourg Strasbourg France; ^12^ Département de Médecine Translationnelle et Neurogénétique Institut de Génétique et de Biologie Moléculaire et Cellulaire, INSERM‐U964/CNRS‐UMR7104/Université de Strasbourg Illkirch‐Graffenstaden France; ^13^ Fédération de Médecine Translationnelle de Strasbourg Université de Strasbourg Strasbourg France; ^14^ Sorbonne Université INSERM, CNRS, Institut de Biologie Paris Seine, IBPS, Neuroscience Paris Seine, NPS Paris France; ^15^ Institut des Neurosciences de Montpellier INSERM U 1298 Montpellier France

**Keywords:** *ADCY5*‐mosaic, mixed movement disorder *ADCY5* (MxMD*‐ADCY5*), paroxysmal dyskinesias (PxD)

## Abstract

**Background:**

An increasing number of *ADCY5*‐mosaic patients, seemingly with a milder phenotype, are being identified. However, an in‐depth assessment of their clinical characteristics is lacking.

**Cases:**

We collected and analyzed data from 12 consecutive *ADCY5*‐mosaic patients diagnosed at our center and 7 cases from the literature; 63% of the patients presented with a baseline hyperkinetic motor disorder with paroxysmal motor exacerbations; 30% had isolated paroxysmal dyskinesias (PxD). Caffeine treatment was highly effective. Developmental delay was observed in 5 patients and especially in those with persistent motor symptoms. PxD were the initial motor symptom in 70% of cases.

**Conclusions:**

*ADCY5*‐mosaic carriers may have the same phenotypic spectrum as non‐mosaic carriers but with a milder clinical presentation. Isolated PxD with onset in infancy are a red flag for *ADCY5*‐mosaic variants. Particular attention should be paid when genetic analysis of patients with this phenotype is conducted as mosaicism can be easily missed.

Variants in the adenylyl cyclase 5 (*ADCY5*) gene are associated with a mixed movement disorder (MxMD*‐ADCY5*) typically characterized by a complex, childhood‐onset hyperkinetic movement disorder (MD).^1^ *ADCY5* converts adenosine triphosphate (ATP) into cyclic adenosine monophosphate (cAMP) and is predominantly expressed in the striatum, playing a crucial role in regulating movement execution.^2^ MxMD‐*ADCY5* is mostly associated with monoallelic pathogenic variants that may be inherited in an autosomal dominant manner or may occur *de novo*. Biallelic variants with autosomal recessive transmission have sporadically been reported.^3^ Whereas certain monoallelic variants seem to act through a gain‐of‐function mechanism increasing cAMP levels ultimately leading to various hyperkinetic MDs, a loss‐of‐function mechanism has been hypothesized for some biallelic variants.^2^ The prototypical presentation is characterized by a nonprogressive, childhood‐onset baseline hyperkinetic MD associated with axial hypotonia and superimposed paroxysmal motor exacerbations.^3^, ^4^ Baseline MDs include chorea, dyskinesias, and dystonia and myoclonus, usually combined. Paroxysmal dyskinesias (PxD) can be the symptom of onset and mainly consist of exacerbations of choreic and/or dystonic movements lasting seconds to hours, and occurring multiple times per day.^3^, ^4^ Nocturnal PxD and perioral jerks are considered hallmarks of the disease and represent key diagnostic clues.^3^, ^4^ Developmental delay (DD) is reported in most patients, possibly affecting both motor and language development, although cognition is usually preserved.^3^, ^4^ Caffeine seems to be a safe and effective treatment for both baseline MDs and paroxysmal episodes,^5^ probably acting as an adenosine 2A receptor (A_2A_R) antagonist and decreasing cAMP levels in the striato‐pallidal neurons.^5^ Other A_2A_R antagonists, such as theophylline or istradefylline, may constitute an alternative treatment for MxMD‐*ADCY5* patients.^6^ Furthermore, benzodiazepines have proven to be helpful.^3^ Finally, deep brain stimulation can be an effective option for the most severe cases.^3^ Advances in genetic analysis have led to an expansion of the phenotypic spectrum and associated variants, with multiple reports highlighting high intra‐familial variability.^4^ A high prevalence of *ADCY5*‐mosaic patients has emerged as well, accounting for up to 43% in a series of 16 *de novo* cases,^4^ possibly contributing to this variability. Genetic mosaicism refers to the presence of genetically different cell lines within the same individual, increasing the phenotypic complexity and challenging genotype–phenotype correlations. In MxMD‐*ADCY5*, mosaicism has been linked to milder^4^, ^7^, ^8^ or asymptomatic phenotypes,^9^ but phenotypic specificity of this group of patients has not been formally studied. Here we characterize in detail 12 consecutive patients with MxMD‐*ADCY5* and genetic mosaicism (MxMD‐*ADCY5*‐mosaic), diagnosed at the Laboratory of Molecular Genetics of Saint Louis Hospital in Paris over 18 years. Seven additional cases were retrieved from the literature. When possible, we compared the clinical phenotype of patients with that of their non‐mosaic affected relatives.

## Case Series

The patients' clinical characteristics are summarized in Table S1.

Clinical information was retrieved from the medical charts of 12 MxMD‐*ADCY5*‐mosaic patients diagnosed in our center. Six of these patients have already been described in a previous publication.^4^, ^5^ A review of the literature conducted on PubMed to identify reports of patients with sufficient clinical data yielded 7 additional cases.^4^, ^7^, ^8^, ^9^, ^10^ All the subjects were symptomatic except for 1 (patient 19), who was identified using genetic analysis performed to assess segregation of a variant detected in her affected daughter.^9^ The age range of included patients varied between 3 and 76 years (mean: 27 years). Mean age at symptom onset was 2.2 years (range: 1–6 years). PxD were the initial motor symptom in 14 cases, preceded by DD in 4 cases. In the remaining subjects, nonparoxysmal MDs were the presenting symptom, manifesting either as a combination of chorea and dystonia for 3—preceded by DD in 1—or as isolated chorea (patient 16). Overall, DD was reported in 5 of 16 cases with available neurodevelopmental history, consistently affecting motor skills and accompanied by language delay in 3. Cognition was reported as normal in 12 subjects, whereas intellectual disability was observed in 3 (patients 4, 11, and 14), although no formal cognitive evaluation was reported in most cases. Psychiatric symptoms were reported in only 2 patients, namely psychosis (patient 15) and behavioral disturbances with low frustration tolerance and suicidal thoughts (patient 11). Persistent baseline hyperkinetic MDs were present in 12 cases, including chorea (8/18), dystonia (10/18), and myoclonus (2/18), isolated but most often combined. All the patients could walk unaided at last follow‐up. Six patients presented with isolated PxD. Overall, 17 of 19 patients presented with PxD, usually with a combination of nocturnal and daytime dyskinetic episodes (13/17). Nocturnal episodes were reported in 15 of 19 cases. Daytime dyskinesias could present as paroxysmal kinesigenic dyskinesias, paroxysmal exercise‐induced dyskinesias, or paroxysmal non‐kinesigenic dyskinesias. Dyskinetic episodes were associated with pain in 5 patients. Other common associated features included facial dyskinesias (8/18) and axial hypotonia (9/18). Pyramidal signs were observed only in patients 15 and 11. Brain magnetic resonance imaging was available for 7 patients and was normal in 6 of these, whereas patient 7 presented corpus callosum hypoplasia. Nine subjects received caffeine treatment, with high efficacy reported for 8. In patient 11, caffeine was only partially effective as he developed recurrent status dystonicus eventually requiring deep brain stimulation, although important recrudescence of paroxysmal motor spells occurred when he tried to suspend caffeine supplementation. Genetic analysis revealed a c.1252C>T/p.R418W variant in 13 subjects. Other detected variants included c.3086 T>A/p.M1029K (patient 13), c.1252G>A/p.R418Q (patient 4), c.1252C>G/p.R418G (patient 17), c.2088 + 1G>A (patient 5), c.2722G>A/p.E908K (patient 19), and c.2088 + 1G>T (patient 7). Mosaic rate information was available for 14 patients and varied between 2.5% and 25%. For the patients diagnosed at our laboratory, low‐level mosaic variants were confirmed using allele‐specific polymerase chain reaction. Clinical data of non‐mosaic affected relatives were accessible for patients 13, 1, 16, 17, and 19 (Table S2). The daughter and nieces of patient 13 had a more severe phenotype compared with her, mainly characterized by an invalidating choreo‐dystonic baseline MD rendering them wheelchair bound.^4^ The daughter of patient 1,^4^, ^5^ the son of patient 16,^8^ and the daughter of the asymptomatic case (patient 19)^9^ had a more severe presentation compared with their mosaic parents. Conversely, the son of patient 17^10^ presented a less‐severe phenotype compared with his mosaic father, with a mild choreo‐dystonic MD without paroxysmal exacerbations.

## Discussion

Patients with mosaicism tend to have a less‐severe phenotype than non‐mosaic *ADCY5* patients^4^, ^5^: (1) they are less likely to present baseline hyperkinetic movements (63% vs. 90%), and (2) they almost always have milder manifestations compared to their non‐mosaic offspring. Interestingly, early‐onset isolated PxD, whether preceded by DD or not, were the initial motor manifestation in 70% of the patients with mosaicism. Of note, in 1 family, the father with mosaicism exhibited a more severe phenotype than his non‐mosaic affected son.^10^ This might suggest that all, or most of, the striatal neurons of the father carry the pathogenic variant despite the observed mosaicism in blood cells. In the nervous system, *ADCY5* is mainly expressed in the striatum and olfactory tubercle, and its critical role in striatal projection neurons likely explains the prominent motor symptoms observed in this condition.^1^ Even in patients with low‐level mosaicism, striatal expression of the pathogenic variant may be sufficient to trigger clinical manifestations, accounting for the similar motor symptoms observed in patients with or without mosaicism. Conversely, we speculate that in the asymptomatic carrier^9^ the pathogenic variant may have been absent (or present at a low level) in the striatal neurons, whereas a germline or mixed gonadal‐somatic variant was likely transmitted to her affected child. As most mosaic subjects present a milder phenotype, other factors yet to be elucidated must play a role in determining clinical severity; 68% of cases had the c.1252C>T/p.R418W variant, which is consistent with the previous observation that position 418 of the gene is a mutational hot spot in patients without mosaicism. In an international multicenter series, *ADCY5*‐mosaic patients represented 43% (7/16) of *de novo* cases.^4^ Interestingly, more than half of them^4^ were French, possibly indicating a higher tendency to look for mosaic variants in our center. In our laboratory, *ADCY5*‐mosaic patients constitute 55% of all subjects identified with an *ADCY5* variant and 2% of all cases genetically analyzed for a clinical presentation of PxD. Detecting mosaic variants, especially with a low variant load, mainly depends on the technical and analytical tools used. The callers used must be capable of detecting mosaicism, whereas filters should not eliminate variants with a low variant allele frequency (VAF). To detect germline variants in a diagnostic context, the classically used parameters are a minimum coverage of 30× and a 5% VAF threshold. Applying these parameters, for a 5% mosaicism and a 30× coverage, the mutated variant would be detected in only 1 or 2 reads. Low‐level *ADCY5‐*mosaics are therefore likely to be missed, due to either a low mosaic ratio or a small number of mutated reads. For *ADCY5*, it is extremely important to use appropriate analysis parameters (minimum gene coverage >100× and 3% VAF threshold) to detect low‐rate mosaicism, with particular attention to the analysis of the mutational hot spots Arg418 and c.2088 + 1G>A.

In conclusion, the presence of PxD, whether isolated or associated with DD, should raise suspicion of *ADCY5‐*mosaicism, especially with onset in infancy or early childhood and/or with nocturnal occurrence. Diagnosis using an adequate approach is particularly important in this treatable disease, especially as caffeine is a safe but not routinely used treatment for hyperkinetic MDs.

## Author Roles

(1) Research project: A. Conception, B. Organization, C. Execution; (2) Statistical analysis: A. Design, B. Execution, C. Review and critique; (3) Manuscript preparation: A. Writing of the first draft, B. Review and critique.

A.I.: 1B, 1C, 2A, 2B, 3A

E.R.: 1A, 1B, 2C, 3A

F.R.: 1A, 1B, 1C, 3A

M.‐C.F.‐H.:1A, 1B, 3B

B.L.: 1B, 1C, 3B

G.G.: 1B, 1C, 3B

M.C.: 1B, 1C, 3B

E.C.‐P.: 1B, 1C, 3B

A.‐C.C.: 1B, 1C, 3B

M.‐A.S.: 1A, 1B, 3B

B.D.: 1B, 1C, 3B

C.S.: 1A, 1B, 3B

P.D.: 1A, 1B, 3B

T.W.: 1A, 1B, 3B

M.A.: 1A, 1B, 3B

E.R.: 1A, 1B, 3B

E.C.: 1A, 1B, 3B

C.D.: 1A, 1B, 3B

O.T.: 1A, 1B, 3B

A.M.: 1A, 1B, 2C, 3A

A.R.: 1A, 1B, 2C, 3A

## Disclosures

**Ethical Compliance Statement:** This study was carried out in accordance with the Declaration of Helsinki and was approved by the local ethics committee (ADYC5MO‐CP‐2024‐08‐112). Written informed consent for scientific publication of the patients' data was obtained from the patients or their legal guardians. We confirm that we have read the journal's position on issues involved in ethical publication and affirm that this work is consistent with those guidelines.

**Funding Sources and Conflicts of Interest:** This study was supported by ADCY5.org. The authors declare that there are no conflicts of interest relevant to this work.

**Financial Disclosures for the Previous 12 Months:** A.I., F.R., M.‐C.F.‐H., B.L., G.G., M.C., A.‐C.C., M.‐A.S., B.D., M.A., E.R., E.C., and C.D. report no financial disclosures for the previous 12 months. E.R. reports honorarium for speech from Orkyn, Aguettant, Elivie, Merz‐Pharma, Janssen, and Teva and for participating in advisory boards from Merz‐Pharma, Elivie, Teva, and Bial. He received research support from Merz‐Pharma, Orkyn, Elivie, Everpharma, Amadys, Aguettant, ADCY5.org, Fonds de dotation Patrick Brou de Laurière, Agence Nationale de la Recherche, Dystonia Medical Research Foundation, Hope for Annabel, Cure Alternating Hemiplegia of Childhood Alternating Hemiplegia of Childhood Foundation, Alternating Hemiplegia of Childhood Association of Iceland, Association française de l'hémiplégie alternante, and Alternating Hemiplegia of Childhood Kids of the Netherlands. E.C.‐P. reports a study grant from the Switzerland‐based Fondation Ancrage for her clinical and research fellowship in abnormal movements in Montpellier, France. C.S. reports honoraria for advisory boards from Egetis, Italfarmaco, Novartis, and Argenx and for speech from Biogen and Lupin. P.D. reports honorarium for speech from AbbVie, Teva, and Novartis and participating in the advisory board for Teva. T.W. reports honoraria from IPSEN, AbbVie, and Edimark; research grants from the Revue Neurologique, the Fondation Planiol, the APTES, and the France Parkinson organizations; prize from the Société Française de Neurologie; and travel funding from LVL Medical, the Movement Disorders Society, Homeperf, and Lündbeck. A.M. reports honoraria for speeches from Merz‐Pharma and travel funding from Elivie and Merz‐Pharma. A.R. reports research support from PTC and travel funding from PTC and Biomarin.

## Supporting information


**Table S1.** Clinical characteristics of the patients.
**Table S2.** Comparison between mosaic patients and their non‐mosaic relatives.

## Data Availability

The data that support the findings of this study are available on request from the corresponding author. The data are not publicly available due to privacy or ethical restrictions.
