# Supplementary material for: ADCY5 ‐Mosaic Variants: A Diagnosis Not to Be Missed
Source: Mov Disord Clin Pract. 2025 Jun 12;12(11):1968–71. doi: 10.1002/mdc3.70175 (PMC12625108; doi:10.1002/mdc3.70175)
Supplement: Supplementary file 1 — Table S1. Clinical characteristics of the patients. Table S2. Comparison between mosaic patients and their non‐mosaic relatives. [file MDC3-12-1968-s001.docx]

**Supplementary Table 1. Clinical characteristics of the patients**

| Patient ID  /ID previous article | Sex,  Age*  (yrs) | Symptom & age of onset  (yrs) | DD | Gait | Baseline MD | N-PxD | D-PxD | Pain with PxD | Caffeine efficacy | Variant  (mosaic rate, sample analyzed) | Gene coverage &  VAF |
| --- | --- | --- | --- | --- | --- | --- | --- | --- | --- | --- | --- |
| Pt. 01  /ID019B^4^-Pt.3^5^ | M,76 | PxD  (6) | No | U | dystonia | Yes | Yes | Yes | effective | c.1252C>T  /p.R418W  (blood) | Mean coverage>400X;  VAF 3% |
| Pt. 02  /ID020^4^ | M,7 | chorea, dystonia  (4) | / | U | chorea, dystonia | Yes | Yes | No | / | c.1252C>T  /p.R418W  (4%, blood) | Mean coverage>400X;  VAF 3% |
| Pt. 03  /ID021^4^-pt.14^5^ | M,20 | Pxd  (1.3) | No | U | dystonia,  myoclonus | Yes | Yes | Yes | effective | c.1252C>T  /p.R418W  (14%, blood) | Mean coverage>400X;  VAF 3% |
| Pt. 04  /ID02^4^ | M,18 | PxD  (1.5) | / | U | No | No | Yes | No | / | c.1253G>A  /p.R418Q  (5%, blood) | Mean coverage>400X;  VAF 3% |
| Pt. 05  /Pt.6^5^ | M,11 | PxD  (3) | No | U | fleeting choreo-dystonic movments | Yes | Yes | No | effective | c.2088+1G>A  (6%, blood) | Mean coverage>400X;  VAF 3% |
| Pt. 06  /pt.16^5^ | F,7 | PxD  (1) | No | U | dystonia | Yes | Yes | No | effective | c.1252C>T  /p.R418W  (4%, blood) | Mean coverage>400X;  VAF 3% |
| Pt. 07 | F,3 | PxD  (1.5) | No | U | No | Yes | Yes | No | effective | c.2088+1G>T  (12%, blood) | Mean coverage>400X;  VAF 3% |
| Pt.08 | M,3 | DD(1)/PxD  (1) | M | U | chorea | Yes | Yes | Yes | effective | c.1252C>T  /p.R418W  (5%, blood) | Mean coverage>400X;  VAF 3% |
| Pt. 09 | F,9 | DD(1)  /PxD (3) | M | U | / | No | Yes | No | / | c.1252C>T  /p.R418W  (11%, blood) | Mean coverage>400X;  VAF 3% |
| Pt.10 | F,5 | PxD  (1) | No | U | No | Yes | Yes | No | / | c.1252C>T  /p.R418W  (5%, blood) | Mean coverage>400X;  VAF 3% |
| Pt. 11 | M,15 | DD (1)  /PxD (2) | M+L | A-W | chorea, dystonia, | Yes | Yes | Yes | effective | c.1252C>T  /p.R418W  (18%, blood) | Mean coverage>400X;  VAF 3% |
| Pt.12 | F,39 | PxD  (2) | No | U | No | Yes | Yes | No | effective | c.1252C>T  /p.R418W (2.5%, blood) | Mean coverage>400X;  VAF 2% |
| Pt. 13  /ChDys II-2^4^ | F,70 | PxD  (2) | No | U | chorea, dystonia | Yes | Yes | No | / | c.3086T>A  /p.M1029K  (blood, skin, and fibroblast cells) | / |
| Pt. 14  /ID027^4^ | F,20 | DD (infancy)  /PxD  (5) | M | U | dystonia,  myoclonus | Yes | Yes | No | / | c.1252C>T  /p.R418W  (blood) | / |
| Pt.15  /Ch.4^4^ | M,42 | chorea, dystonia  (<20) | / | U | chorea, dystonia | No | No | No | / | c.1252C>T  /p.R418W  (blood) | / |
| Pt. 16  /sub II-1^8^ | M,67 | Chorea  (1) | No | U | chorea | Yes | No | No | / | c.1252C>T  /p.R418W  (8%, blood) | / |
| Pt. 17  /pt.4^10^ | M,47 | DD (infancy) / chorea, dystonia  (3) | M+L | U | chorea, dystonia | Yes | Yes | Yes | / | c.1252C>G  /p.R418G  (blood) | / |
| Pt.18  /pt.1^7^-Pt.29^5^ | M,32 | PxD  (4) | No | U | No | Yes | No | No | effective | c.1252C>T  /p.R418W  (15%, blood and skin) | Gene coverage: 85X |
| Pt. 19  /parent case 1^9^ | F,40 | asymptomatic | No | U | No | No | No | No | / | c.2722G>A  /p.E908K  (25%, blood) | / |

Legend: F: female; M: male; Age*: current age or when last reported; PxD: paroxysmal dyskinesia; N-PxD: nocturnal PxD; D-PxD: diurnal PxD; DD: developmental delay; M: motor; L: language; U: unassisted; A: assisted; W: wheelchair-bound; VAF: variant allele frequency

**Supplementary Table 2. Comparison between mosaic patients and their non-mosaic relatives**

| Patient ID  / ID in previous articles | Sex,  Age*  (yrs) | Symptom & age of onset  (yrs) | DD | Gait | Baseline MD | N-PxD | D-PxD | Pain with PxD | Caffeine efficacy | Variant  (Mutant load) |
| --- | --- | --- | --- | --- | --- | --- | --- | --- | --- | --- |
| Pt. 01  /ID019B^4^-Pt.3^5^ | M,76 | PxD  (6) | No | U | dystonia | Yes | Yes | Yes | effective | c.1252C>T  /p.R418W  (blood) |
| Daughter pt.01  /ID19^4^-pt.4^5^ | F,40 | / | No | U | dystonia, myoclonus, chorea, dysarthria | Yes | Yes | Yes | effective | c.1252C>T  /p.R418W  (blood) |
| Pt. 13  /ChDys II-2^4^ | F,70 | PxD  (2) | No | U | chorea, dystonia | Yes | Yes | No | / | c.3086T>A  /p.M1029K  (blood, skin, and fibroblast cells) |
| Daughter pt.13  / ChDys III-6^4^ | F,46 | DD (infancy) /chorea (4) | M | W | chorea | No | Yes | Yes | / | c.3086T>A  /p.M1029K  (blood) |
| Niece Pt.13  / ChDys IV-4^4^ | F,22 | DD (<1)/ chorea, dystonia  (8) | M | Not acquired | chorea, dystonia | / | / | / | / | c.3086T>A  /p.M1029K  (blood) |
| Niece Pt.13  / ChDys IV-5^4^ | F,20 | DD (0.5)/ chorea, dystonia  (9) | M | W | chorea, dystonia | / | / | / | / | c.3086T>A  /p.M1029K  (blood) |
| Pt. 16  /sub II-1^8^ | M,67 | chorea  (1) | No | U | chorea | Yes | No | No | / | c.1252C>T  /p.R418W  (8%, blood) |
| Son pt.16  /Sub III-1^8^ | M,36 | chorea  (1) | No | unsteady | chorea | Yes | / | Yes | not effective | c.1252C>T  /p.R418W  (blood) |
| Pt. 17  /pt.4^10^ | M,47 | DD (infancy) / chorea, dystonia (3) | M+L | U | chorea, dystonia | Yes | Yes | Yes | / | c.1252C>G /p.R418G  (blood) |
| Son Pt.17  /pt.3^10^ | M,3 | Chorea  (<1) | M+L | U  (Tiptoe walking) | chorea, dystonia | No | No | No | / | c.1252C>G/p.R418G  (blood) |
| Pt. 19  /parent case 1^9^ | F,40 | asymptomatic | No | U | No | No | No | No | / | c.2722G>A/p.E908K  (25%, blood) |
| Daughter Pt. 19  /Case 1^9^ | F,40 | DD (infancy)/ unspecified painfull movements  (34) | M | paraparetic/dystonic | spasticity, dystonia | / | / | Yes | / | c.2722G>A/p.E908K  (blood) |

Legend: F: female; M: male; Age*: current age or when last reported; PxD: paroxysmal dyskinesia; N-PxD: nocturnal PxD; D-PxD: diurnal PxD; DD: developmental delay; M: motor; L: language; U: unassisted; A: assisted; W: wheelchair-bound
